# PD-1 expression in tumor infiltrating lymphocytes as a prognostic marker in early-stage non-small cell lung cancer

**DOI:** 10.3389/fonc.2024.1414900

**Published:** 2024-09-26

**Authors:** Asaf Dan, Ozan Aricak, Konstantinos Rounis, M. Angeles Montero-Fernandez, Ricardo Guijarro, Simon Ekman, Cristian Ortiz-Villalón, Luigi De Petris

**Affiliations:** ^1^ Department of Oncology-Pathology (Onkpat), Karolinska Institutet, Stockholm, Sweden; ^2^ Thoracic Oncology Center, Karolinska Comprehensive Cancer Center, Stockholm, Sweden; ^3^ Department of Clinical Pathology and Cancer Diagnostics, Karolinska University Hospital, Huddinge, Sweden; ^4^ Department of Laboratory Medicine, Division of Pathology, Karolinska Institutet, Huddinge, Sweden; ^5^ Department of Cellular Pathology, Royal Liverpool University Hospital National Health Service Foundation Trust (NHS FT), Liverpool, United Kingdom; ^6^ Department of Thoracic Surgery, Valencia University, Valencia, Spain

**Keywords:** early-stage NSCLC, TILs, PD-1, prognosis, immunotherapy, Tissue microarray

## Abstract

**Introduction:**

Programmed death ligand – 1 (PD-L1) expression is a well-established predictive biomarker for immunotherapy in non-small cell lung cancer (NSCLC). Programmed death – 1 (PD-1) serves as the target protein to PD-L1 and their interaction serves as a crucial pathway for immune evasion. This study aimed to investigate the expression pattern of PD-1 on Tumor-infiltrating lymphocytes (TILs) in early-stage NSCLC, and its potential role as prognostic biomarker.

**Materials & methods:**

PD-1 was evaluated in 474 surgical resected early-stage NSCLC specimens, using Tissue microarray and immunohistochemical staining. Expression was scored as negative (<1%) or positive. Positive PD-1 expression was further divided into low (<10%) and high (≥10%). None of the patients had received treatment with PD-1/PD-L1 inhibitors.

**Results:**

PD-1 expression ≥1% in TILs was observed in 83.5% of cases and was associated with pT stage (p=0.02), grade 3 (p=0.004), and adenocarcinoma subtype (p=0.05). Individuals with high PD-1 expression (≥10%) experienced reduced 10-year overall survival (Log-Rank test = 0.005). In addition, high PD-1 expression emerged as an independent factor associated with reduced survival on multivariate analysis (HR: 1.328 (95% CI: 1.074-1.641).

**Conclusions:**

Patients with early-stage NSCLC who exhibited PD-1 expression of ≥10% on TILs had an unfavorable 10-year OS rate. These findings indicate that elevated PD-1 expression on TILs can be associated with immune evasion during the early stages of malignancy evolution in the NSCLC setting and further research is required to further delineate the role of PD-1/PD-L1 pathway on tumor immune senescence. These results underline the potential role of PD-1/PD-L1 inhibitors in the treatment of early-stage NSCLC.

## Introduction

Non-small cell lung cancer (NSCLC) represents the cancer subtype associated with the highest mortality on a global scale by 2022 (1). Immunotherapy, with the administration of programmed death-1 (PD-1) or programmed death – 1 ligand (PD-L1) inhibitors, has revolutionized the management of non-small cell lung cancer (NSCLC) that does not harbor specific targetable genomic alterations, offering the possibility of long-term remission in a subset of patients (2).

PD-1 [cluster of differentiation (CD)297] is a cell surface receptor which is upregulated on T cells upon activation through both the T-cell antigen receptor and cytokine receptors. PD-1 is mainly expressed on mature T cells, but it can be also found on a plethora of activated cells of the immune system such as natural killer T cells, B cells, monocytes, immature Langerhans’ cells and CD4-CD8- thymic T cells (3). PD-1 ligands are PD-L1 and PD-L2. PD-L1 is constitutively expressed at low levels on a wide range of antigen-presenting cells (APCs) and also on a variety of non-hematopoietic cell types whereas PD-L2 is expressed on dendritic cells and macrophages post-activation. The expression kinetics of PD-1 expression upon activation suggest that the upregulation of PD-1 is an intrinsic outcome of T-cell activation, serving a crucial role in orchestrating the timely termination of the immune response (3). The aforementioned hypothesis is further reinforced by the fact that PD-1^-/-^ mice develop a plethora of autoimmune phenomena (4).

Evasion of the host’s immune system is one of the hallmarks of cancer (5). Malignancies have the potential to hijack the PD-1/PD-L1 pathway towards their further progression and evolution (6). Furthermore, the constitution and function of the microenvironment of tumors are dynamic processes, as several experimental data demonstrate the stepwise evolution of the tumor microenvironment during the disease trajectory towards the goal of immune evasion and cancer progression (7).

In the pivotal phase III trials that led to the approval of PD-1/PD-L1 inhibitors as treatment for metastatic NSCLC, high expression levels of PD-L1 in the cancer cells or the immune cells of the tumor microenvironment was predictive for treatment effect (8–12). Despite the established role of PD-L1 expression as a predictive biomarker there had been very few data at the time of the conceptualization of our study on the effect of PD-1 expression on tumor-infiltrating-lymphocytes (TILs) (13). In addition, there has been a paucity of data on the prognostic role of PD-1 expression on TILs in early-stage NSCLC and its potential role as a means to further malignant evolution and immune evasion.

In a previous study, we investigated the tumor expression and prognostic role of PD-L1, assessed by four different antibodies, on a cohort of surgically resected early-stage NSCLC (14). In the present study, we (14) evaluate the PD-1 expression on TILs on the same early-stage NSCLC cohort in relation to different clinicopathological characteristics and overall survival (OS).

## Materials and methods

### Study cohort

A total of 598 patients, who underwent curative surgery for NSCLC between 1987 and 2004 at Karolinska University Hospital, Sweden, were initially selected for this study (Ethical approvals No. 2005/588-31/4 – 2008/136-32). None of the patients had received peri-operative anti-PD-1/L1 treatment. Archival formalin fixed paraffin embedded (FFPE) specimens were obtained, where the surgical specimens had undergone routine clinical diagnostic processing at the department of pathology. The clinical data was collected from clinical records and pathology reports. Upon revision, patients with histologically verified NSCLC with early pathological TNM stage (15) were included, excluding neuroendocrine tumors, pulmonary metastases and late-stage NSCLC cases. Furthermore, patients with incomplete clinical data were excluded. The final cohort number was reduced to 474 patients after further excluding cases with damaged or technically unsatisfactory stained TMA cores. A flowchart of the exclusion process and details on patients with unsatisfactory staining is available in **Supplementary Figure S1**.

### Histologic types and grading

The cases were classified by their histologic type, according to the 2021 WHO classification of lung tumors (16). The histologic types selected for this study were adenocarcinoma (AdCa), squamous cell carcinoma (SqCC) and large cell carcinoma (LCC). Adenosquamous tumors (AdSq) were, upon revision, reclassified as adenocarcinoma with squamous differentiation. All cases were re-staged according to the 8^th^edition of the UICC/AJCC staging system (15). The tumors were graded on a three-tier grading system based on architectural and nuclear morphology, similar to the grading suggested in the 2015 WHO classification of lung tumors. Grade 1 (G1) being well differentiated, grade 2 (G2) moderately differentiated, grade 3 (G3) poorly differentiated. In the adenocarcinoma histologic group, G1 tumors were lepidic, G2 were acinar and papillary tumors, G3 tumors were solid and micropapillary tumors. The squamous cell carcinomas were stratified into G1-G3 based on architecture and nuclear atypia. Large cell carcinomas are per definition poorly differentiated tumors (16).

### TMA construction and immunohistochemistry

Tissue microarrays (TMA) were produced by extracting cores measuring 1 mm in diameter from the donor blocks. To account for possible tumor heterogeneity, one core was sampled from the center of the tumor and another from the periphery; resulting in 20 TMA blocks. From each TMA block, 4 µm thick sections were stained with anti PD-1 antibody, validated for routine clinical diagnostic work (according to the manufacturers’ standard protocols (PD-1 clone NAT105, produced by Cell Marque Corporation USA, stained on Ventana Ultra Benchmark platform).

### PD-1 assessment in TILs

Two experienced pathologists reviewed the TMA slides, scoring PD-1 expression in TILS in each of the two cores and the higher percentage score was considered as a representative of the case. If one of the cores was missing or damaged, the remaining one was used for the scoring. Then, for each case, an average of the reviewers’ scores was used as a final PD-1 expression for each case. Only sections containing more than 100 tumor cells were considered to be representative for assessment (17).

**Figure 1:**
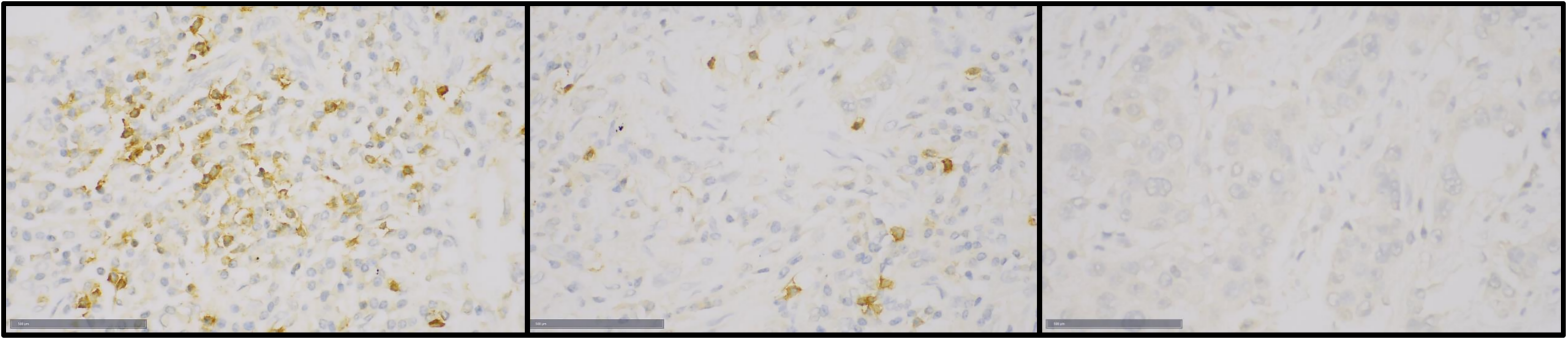
Immunohistochemical staining of PD-1 in TILs of tumor samples in the TMA: Left- high expression (≥10%), center- low expresison (<10%), right- negative (<1%).

A three-tiered system of scoring was applied to record PD-1 expression in TILs; negative (<1% positive TILs), low expression (<10% positive TILs) and high expression (≥10% positive TILs), as depicted in **Figure 1**.

In the absence of a universally accepted cut-off for PD-1 expression in lung cancer, we initially attempted to establish a threshold using Receiver-operating characteristic curve (ROC) analysis. However, this approach did not yield a definitive cut-off. As a result, we selected an arbitrary threshold of ≥10% to categorize PD-1 expression as “high.” To validate this cut-off, we examined the distribution of patients in the <10% and ≥10% groups. Notably, the groups were of comparable size, with approximately 50% of the study population in each group (**Table 1**). The balanced distribution supports the appropriateness of our chosen cut-off.

**Table 1:** Clinicopathological correlation with PD-1 staining.

			PD-1					
n = 474 patients		% of cohort	PD-1 Negative (<1%),	PD-1 Positive (≥1%),	p-value	PD-1 Low (< 10%)	PD-1 High (≥ 10%)	p-value
Total cohort			78 (16.5%)	396 (83.5%)		238 (50.2%)	236 (49.8%)	
10-year OS	Entire cohort: 26%		34%	29%		35%	25%	
Median OS			66 months	74 months	^1^0.98	84 months	62 Months	**^1^0.005**
Age (39-86)	<70 (n=267)	56.3 %	47 (9.9%)	220 (46.4%)	^2^0.444	141 (29.7%)	126 (26.6%)	^2^0.199
Median= 68	≥70 (n=207)	43.7 %	31 (6.5%)	176 (37.1%)		97 (20.5%)	110 (23.2%)	
Gender	Male (n=262)	55.3 %	46 (9.7%)	216 (45.6%)	^2^0.472	133 (28.1%)	129 (27.2%)	^2^0.789
	Female (n=212)	44.7 %	32 (6.8%)	180 (38%)		105 (22.2%)	107 (22.6%)	
Smoking (n=425) 90%	Current (n=253)	53.4%	33 (7%)	220 (46.4%)	**^2^0.048**	113 (23.8)	140 (29.5%)	**^2^0.016**
	Former (n=133)	28.1%	22 (4.6%)	111 (23.4%)		77 (16.2%)	56 (11.8%)	
	Never (n=39)	8.2%	11 (2.3%)	28 (5.9%)		24 (5.1%)	15 (3.2%)	
Histology	AdCa (n=285)	60.1 %	56 (11.8%)	229 (48.3%)	^2^0.054	157 (33.1%)	128 (27%)	^2^ **0.033**
	SqCC (n=158)	33.3 %	17 (3.6%)	141 (29.7%)		68 (14.3%)	90 (19%)	
	LCC (n=31)	6.5 %	5 (1.1%)	26 (5.5%)		13 (2.7%)	18 (3.8%)	
pT (TNM8)	pT1 (n=175)	36.9 %	38 (8%)	137 (28.9%)	^2^ **0.018**	94 (19.8%)	81 (17.1%)	^2^0.243
	pT2-T3 (n=299)	63.1 %	40 (8.4%)	259 (54.6%)		144 (30.4%)	155 (32.7%)	
Stage (TNM8)	IA (n=224)	47.3 %	47 (9.9%)	177 (37.3%)	^2^0.061	117 (24.7%)	107 (22.6%)	^2^0.466
	IB (n=146)	30.8 %	21 (4.4%)	125 (26.4%)		76 (16%)	70 (14.8%)	
	IIA (n=49)	10.3 %	5 (1.1%)	44 (9.3%)		22 (4.6%)	27 (5.7%)	
	IIB (n=55)	11.6 %	5 (1.1%)	50 (10.5%)		23 (4.9%)	32 (6.8%)	
Grade	G1 (n=91)	19.2 %	21 (4.4%)	70 (14.8%)	^2^ **0.004**	56 (11.8%)	35 (7.4%)	^2^ **<0.001**
	G2 (n=181)	38.2 %	37 (7.8%)	144 (30.4%)		102 (21.5%)	79 (16.7%)	
	G3 (n=202)	42.6 %	20 (4.2%)	182 (38.4%)		80 (16.9%)	122 (25.7%)	

OS, overall survival; AdCa, Adenocarcinoma; SqCC, squamous cell carcinoma; LCC, Large cell carcinoma; pT, pathological T stadium. ^1^Log-Rank test comparing the analyzed covariates with median overall survival. ^2^Chi-Square test comparing differential frequencies amongst the analyzed covariates. Results with p<0.05 are annotaed with bold font.

### Follow-up time and survival outcomes

Overall survival (OS) was calculated from the date of surgery to death of any cause, or to last follow up. For patients with no disease recurrence after 10 years of follow-up, survival was censored at 122 months. The median follow-up time for censored patients was 122 months, with a minimum follow-up time of 28 months. Disease-free survival was calculated from the date of surgery until evidence of radiological or clinical recurrence, or to death of any cause. In order to evaluate disease-specific survival, we calculated Time-To-Progression (TTP), similarly to DFS, but patients dying without evidence of lung cancer recurrence were censored at last follow up.

### Statistics

All statistical analyses were performed with SPSS 26.0.0 software (IBM Corp., Armonk, NY, USA). Descriptive statistics were applied to define and categorize nominal and categorical variables. Statistical significance was set at the level of p-value <0.05. PD-1 expression was grouped into two sets, positive vs negative, and low/negative vs high expression. These subgroups were then used for further statistical analyses. The association between PD-1 expression and clinicopathologic data was calculated using Fisher’s exact test. The log-rank test was applied to investigate the effect of the analyzed co-variates on 10 years OS. The effect of age ≥ 70 years old, grade 3 tumors (compared with grade 1 or 2), p-stage II and PD-1 expression ≥ 10% on 10 years OS was calculated through the application of univariate Cox regression analysis. The parameters that reached statistical significance in the univariate analysis were further analyzed using multivariate Cox regression analysis.

## Results

### The cohort and tumor characteristics

The detailed characteristics of the patients in our cohort are depicted in **Table 1**. The median age of the individuals was 68 years (range: 39-86 years). Gender distribution was almost equal (male 55%, female 45%). Smoking status was documented for 90% of the patients, with 53.4% identified as current smokers. Most cases were AdCa (60.1%), followed by SqCC (33.3%) and LCC (6.5%). Most of the cases had pathological stage IA-IB (78%) and almost a third of all cases had a tumor stage of pT1 (36.9%). Nearly half of the cases showed a high tumor grade (G3 42.6%), followed by intermediate grade (G2 38.2%). The 10-year OS was 27% for the entire cohort. Only three patients received adjuvant chemotherapy, minimizing its impact on our analysis of PD-1’s prognostic significance. The main site of recurrence was intrathoracic (58 patients, 12% of our cohort). The first site of relapse after operation is specified in **Supplementary Table S1**.

### PD-1 immunohistochemical staining and clinicopathologic data

The majority of cases in our cohort (83.5%) had a PD-1 expression in TILs equal or higher to 1% (**Table 1**). PD-1 ≥ 1% in TILs demonstrated significant association with pT stage (p=0.018), tumor grade (p=0.004) and smoking (p=0.048). PD-1 ≥ 1% was not associated with age group (p=0.444), gender (p=0.472) or 10-year OS (p=0.986) (**Table 1**).

Approximately half of the cases (49.8%) had high PD-1 expression (≥10%) in TILs. PD-1 expression ≥10% in TILs was associated with tumor grade (p<0.001), 10-year OS (p=0.008), histologic group (p=0.033) and smoking (p=0.016) at a statistically significant level (**Table 1**). There was no significant association between high PD-1 expression and age (p=0.199), gender (p=0.789), pT-stage (p=0.243) or pathological TNM stage (p=0.466) (**Table 1**).

### PD-1 as a prognostic factor

**Figure 2:**
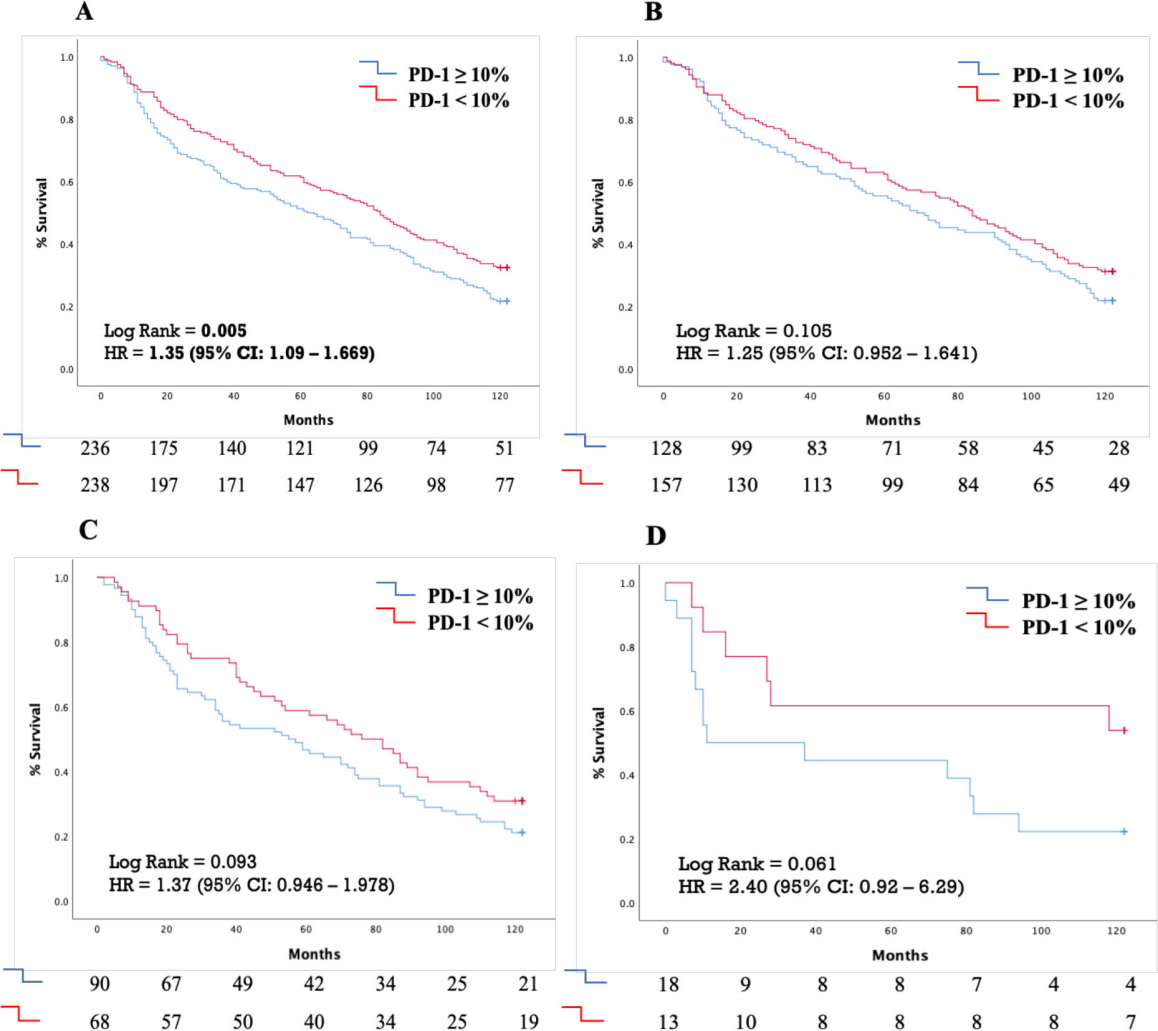
Kaplan-Meyer curves demonstrating the effect of PD-1 expression cut-off of 10% on 10-year survival in **(A)** The whole patient cohort **(B)** Patients with lung adenocarcinoma **(C)** Patients with squamous cell lung carcinoma **(D)** Patients with large cell lung carcinoma. PD-1, Programmed death-ligand 1; HR, Hazard ratio; CI, Confidence intervals.

Individuals with PD-1 expression ≥ 10% in TILs experienced lower 10 years OS in comparison to those with PD-1 expression < 10% (median OS of 62 months vs 84 months, respectively; p = 0.005) (**Figure 2A**). A similar trend for worse outcome in PD1 ≥ 10% positive cases was observed even in the histological subgroups of adenocarcinoma, squamous-cell carcinoma and large-cell carcinoma, although without reaching statistical significance (**Figures 2B–D**, respectively).

Additionally, this trend was evident in tumor stages, with a modest difference in stage I tumors and a more pronounced difference in stage II tumors, where PD-1 ≥ 10% was also associated with a poorer prognosis (median OS of 23 months [95% CI: 7.95–59.32] vs. 71 months [95% CI: 17.11–124.89]) (**Supplementary Figure S3**).

In the univariate Cox Regression analysis age ≥ 70 years old [HR: 1.252 (95% CI: 1.014-1.548), p = 0.037], stage II disease [HR=1.453 (95% CI: 1.133-1.862), p=0.003] and PD-1 expression in TILs ≥ 10% [HR=1.351 (95% CI:1.093-1.669), p=0.005] were associated with diminished 10 years OS (**Table 2**). Individuals with grade 3 tumors did not experience reduced 10 years OS compared to those with grade I or grade II tumors [HR: 1.103 (95% CI: 0.892-1.365), p=0.367]. However, in the multivariate analysis, only stage II disease [HR=1.438 (95% CI: 1.122-1.844), p=0.005] and PD-1 expression in TILs ≥ 10% [HR=1.328 (95% CI: 1.074-1.641), p=0.009] emerged as independent negative prognostic factors for reduced 10 years OS (**Table 2**).

**Table 2:** Univariate and multivariate analysis through the application of Cox Regression on the effect of the age ≥ 70 years old, grade 3 tumors, stage II and PD-1 expression ≥ 10% on TILs on 10 years survival in our patients’ cohort.

Univariate analysis	HR (95%CI)	*p* value
Age ≥ 70 years old	1.252 (1.014-1.548)	**0.037**
Grade 3	1.103 (0.892-1.365)	0.367
Stage IIA or IIB*	1.453 (1.133-1.862)	**0.003**
PD-1 ≥ 10%	1.351 (1.093-1.669)	**0.005**
Multivariate analysis	HR (95%CI)	*p* value
Age ≥ 70 years old	1.231 (0.996-1.522)	0.055
Stage IIA or IIB	1.438 (1.122-1.844)	**0.005**
PD-1 ≥ 10%	1.328 (1.074-1.641)	**0.009**

The variables that achieved statistical significance (p<0.05) in the univariate underwent multivariate analysis.

HR, Hazard ratios; CI, confidence intervals; PD-1, Programmed cell death-1.

*AJCC TNM 8^th^ edition. Results with p<0.05 are annotaed with bold font.

**Figure 3:**
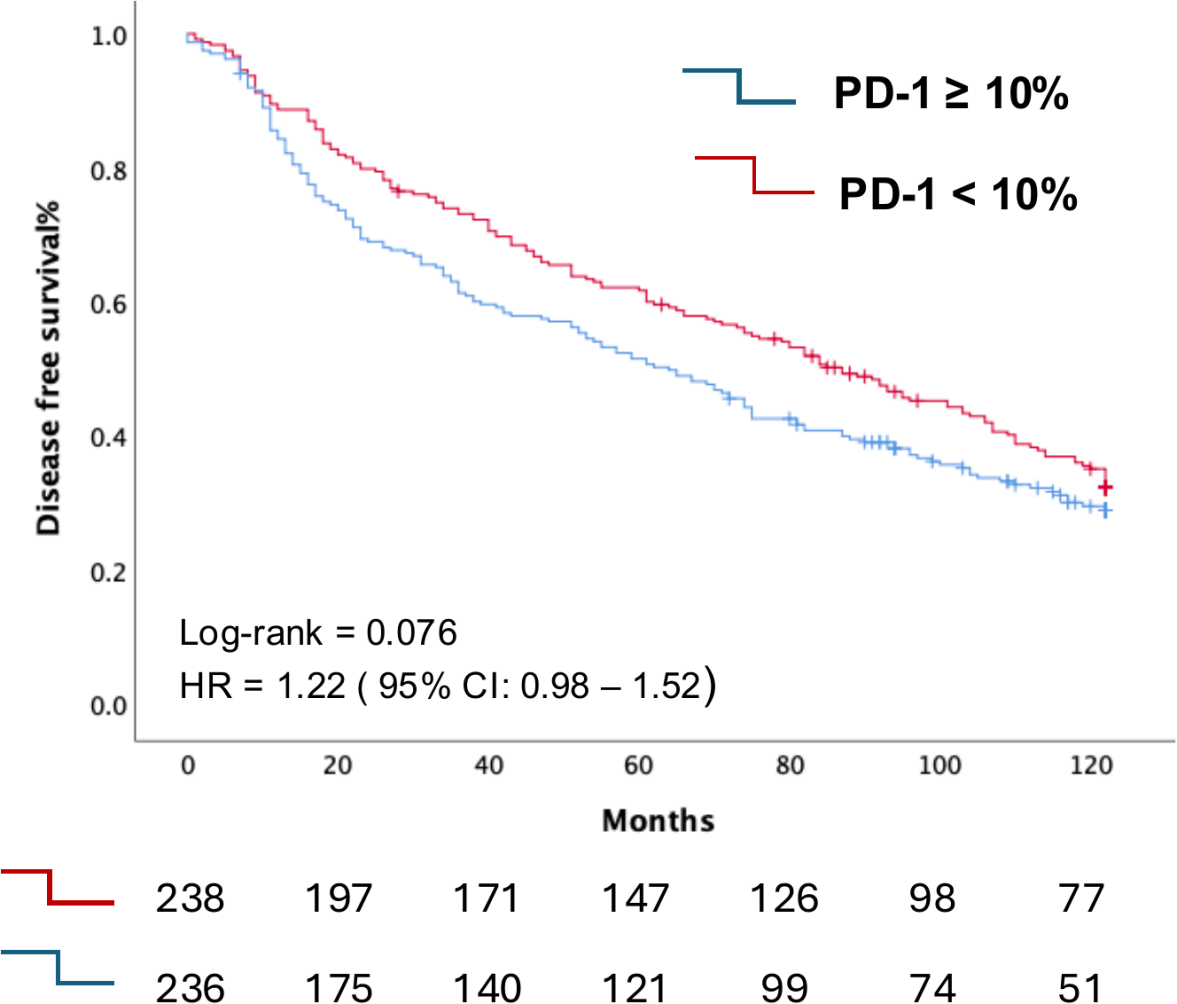
Kaplan-Meier curve depicting the differential Disease-Free Survival curves according to PD1 expression in TIL between individuals in our cohort with PD-1 expression in their T cells <10% compared with those with PD-1 expression ≥10%.

Regarding disease-free survival (DFS) and time to progression (TTP), Kaplan-Meier analysis demonstrated a non-significant trend towards worse DFS in patients with PD-1 expression ≥ 10%, with a median DFS of 64 months (95% CI: 52–77 months), compared to those with PD-1 expression < 10%, who had a median DFS of 87 months (95% CI: 71–103 months) (**Figure 3**).

No differences in TTP were observed between the two groups. The median TTP was not reached for patients with PD-1 expression < 10%. For those with PD-1 expression ≥ 10%, the median TTP was 96 months (95% CI not amenable for calculation). (**Supplementary Figure S2**).

### Association between PD-1 and PD-L1

In our comprehensive analysis, we previously evaluated the clinicopathological significance of four distinct PD-L1 antibodies within the same patient cohort, utilizing different TMA samples (14). Building on this foundation, we specifically investigated the association between PD-1 expression on tumor-infiltrating lymphocytes (TILs) and tumor PD-L1 expression using the PD-L1 antibody clone 28-8, which is a validated assay in clinical routine.

**Figure 4:**
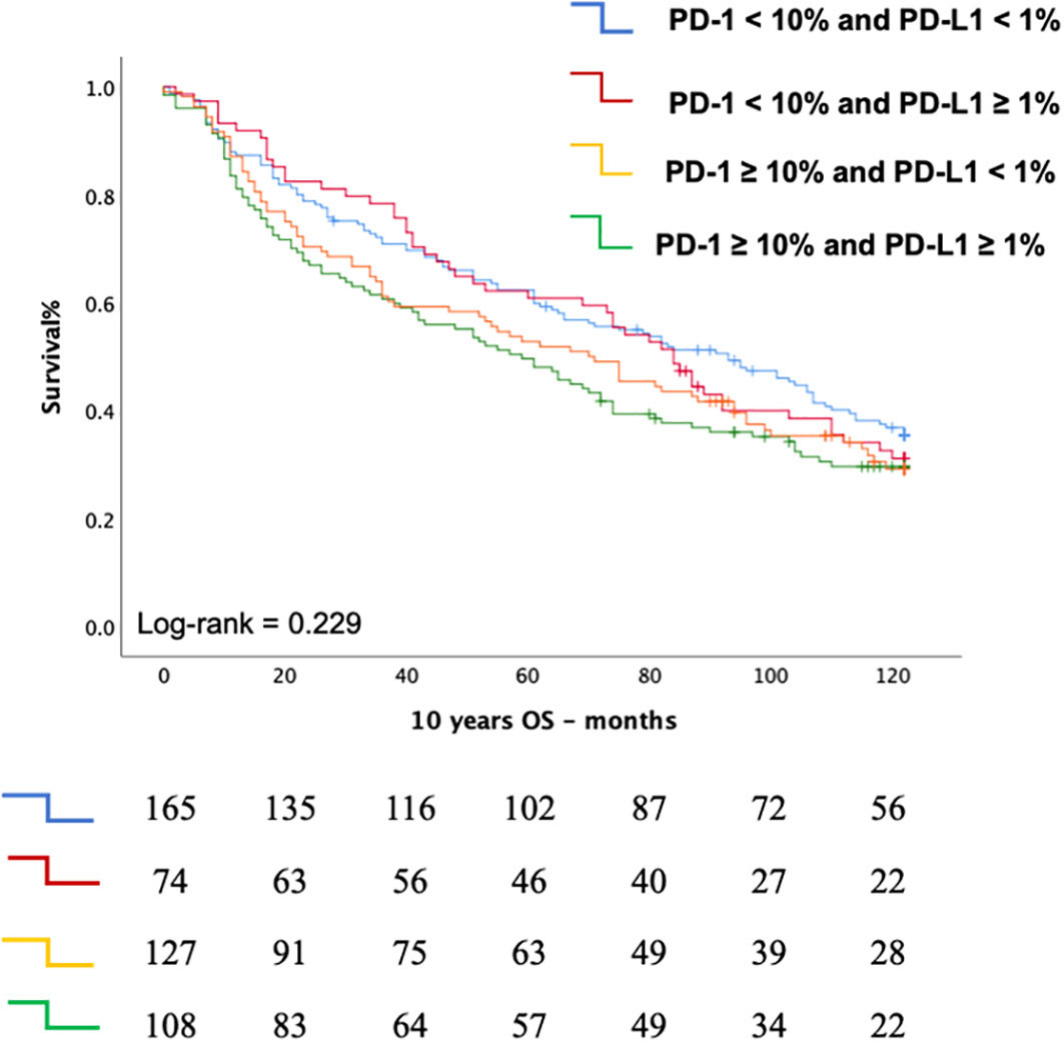
Kaplan Meier curve depicting the Overall Survival (OS according to the combined status of PD-1 expression on T cells and PD-L1 expression on tumor cells.

The Chi-square test revealed a significant association between PD-1 expression levels and PD-L1 expression (p = 0.002) (**Table 3**). Kaplan-Meier survival analysis further highlights the prognostic significance of PD-1, showing that elevated PD-1 levels are associated with comparably poor outcomes in both PD-1 ≥ 10% with PD-L1 ≥ 1% (median OS: 70 months, 95% CI: 47.09–84.74 months) and PD-1 ≥ 10% with PD-L1 < 1% (median OS: 59 months, 95% CI: 42.86–75.14 months). This reinforces the role of PD-1 expression in TILs as a negative prognostic marker (**Figure 4**).

**Table 3:** Chi-square test demonstrating the statistical association of PD-1 expression levels on T cells using a cut-off value of 10% with PD-L1 tumor expression levels.

		PD-L1 < 1%	1% ≤ PD-L1 < 50%	PD-L1 ≥ 50%	Total	p-value
**PD-1 expression on TILs**	**< 10%**	160 (33%)	56 (12%)	22 (5%)	238 (50%)	**0.002**
	**≥ 10%**	125 (26%)	67 (14%)	44 (9%)	236 (50%)	
**Total**		285 (50%)	123 (26%)	66 (14%)	474 (100%)	

Results with p<0.05 are annotaed with bold font.

## Discussion

In our study cohort, High PD-1 expression of ≥10% in TILs in individuals with early-stage NSCLC constituted an independent negative prognostic factor. Our results highlight a subgroup amongst patients with early-stage NSCLC with highest risk of death that might potentially benefit from the intensification of adjuvant therapy by the addition of PD-1/PD-L1 inhibitors.

PD-1/PD-L1/PD-L2 interaction has been a well-established immune pathway associated with the development of immune tolerance (18–20) and the occurrence and sustainment of immune evasion in the setting of malignancy (3). Experimental data have demonstrated that high expression levels of PD-L1 on the surface of tumor cells can be sufficient for the evasion of a robust host’s antitumor immune response (21). Furthermore, high level of TILs, especially CD8^+^ T cells have been associated with favorable outcomes in NSCLC patients undergoing immunotherapy (22, 23). In addition, in NSCLC patients treated with second line atezolizumab, high levels of PD-L1 expression in their TILs was associated with favorable clinical outcomes (12). However, the majority of the published reports on NSCLC have been done in the metastatic setting and there is a relative paucity of data on the early setting or on the specific context of the infiltrating TILS and their expression of specific surface protein markers such as PD-1.

More analytically, several studies have investigated the predictive and prognostic value of TILs in a wide range of malignancies. Edlund et al. (24) reported that T cell, B cell and plasma cell infiltration was associated with better outcome across malignancies, yet not in tumors with high (>50%) PD-L1 expression, which supports the key role of PD-L1 expression in antitumoral response. Schulze et al. (25) reported similar results with high CD8+ T cell infiltration as an independent prognostic factor for favorable overall survival in PD-L1 negative NSCLC but not in PD-L1 positive. Other studies have also reported a favorable prognostic value of increased TILs in NSCLC (26, 27).

The association between PD-1 expression and clinicopathological data, as well as prognosis has been investigated in several different tumor types, with conflicting results. Muenst et al. (28) reported increased PD-1 positivity in TILs as a negative prognostic factor in classical Hodgkin lymphoma, yet Carreras et al. (29) reported improved OS with high PD-1 positivity in TILs in follicular lymphoma and Pollari et al. (30) reported similar findings in primary testicular lymphoma. Owen et al. (31) found no correlation between high PD-1 positivity in TILs and OS in thymic epithelial tumors, although the cohort was limited (n=35) and split into several tumor subtypes.

Comparatively, there has been limited focus on PD-1 expression in TILs in relation to clinicopathological aspects, predictive value and prognosis in NSCLC. Only a handful of studies are available with varying results. Paulsen et al. (32) reported that high PD-1 expression in the subset of intraepithelial TILs was associated with better 5 year disease specific survival (DSS). Koh et al. (33) describe, interestingly, that a high number of PD-1 positive cells (cutoff 25/mm2) was associated with prolonged disease-free survival (DFS), whereas a PD-1+/CD8+ ratio above 0.25 was associated with shorter DFS. Aránzazu Lafuente-Sanchis et al. (34) reported an inverse PD-1 level correlation with shorter DFS and OS. On the other hand, Schmidt et al. (13) did not see any prognostic significance of PD-1 positivity (cutoff at 5%). Deng et al. (35) studied the expression levels of the *PDCD1* gene and found that higher levels of gene expression correlate with worse OS. He et al. (36) have investigated the clinical impact of PD-1 expression levels in TILs in NSCLC from 139 surgical resection specimens, that included clinical stages I-IV. They set PD-1 positivity cutoff at 8% and a 5 year follow up period for OS, in which they did not see a significant correlation between PD-1 expression and OS. These studies have variations in methods, the latter two applying detection methods other than IHC, different scoring systems for PD-1 positivity, varying cutoff levels for statistical analyses and different time span for OS, DSS, DFS and PFS. Any of these differences in methods, as well as a combination of them, may affect the results of each investigation.

Moreover, since the administration of peri-operative immunotherapy is becoming standard of care in a subset of early-stage NSCLC, the issue about validating robust predictive biomarkers in this setting is of high interest. In clinical trials with neo-adjuvant or adjuvant (chemo)-immunotherapy, the favorable effect of immunotherapy was more pronounced in individuals with high PD-L1 expression (37–39), whereas the role of PD-1 has been less extensively investigated (14). However, our findings, alongside previous research, suggest that PD-1 expression, particularly when combined with PD-L1 expression, may offer valuable prognostic insights. In addition, in a retrospective study of 188 individuals with advanced NSCLC treated with immune checkpoint inhibitors, the functional engagement of PD-1/PD-L1, that was visualized through the application of a high-throughput automated quantitative imaging platform, served as a better predictive biomarker than PD-L1 expression levels alone (40). The latter report delineates even more the complexity of the PD-1/PD-L1/PD-L2 pathway and highlights the potential disadvantages of PD-L1 expression as the solely prognostic and predictive biomarker. Our findings further emphasize the importance of considering both PD-1 and PD-L1 expression in this context, as their interaction may more accurately reflect the tumor immune environment and patient outcomes.

In our study we found that PD-1 positivity in TILs was associated with tumor grade and pT stage, and that high (≥10%) PD-1 expression was correlated with histology and tumor grade. Univariate and multivariate regression analyses support high PD-1 expression in TILs as an independent negative prognostic factor in this cohort. A similar trend was observed in all histological subgroups, and the lack of statistical significance can be attributed to a statistical type II error.

Nevertheless, our results are in accordance with some of the similar reports described above.

To our knowledge this study is one of the few studies that investigate the effect of PD-1 levels on TILs on a large patient cohort and follow-up time >10 years, and the first one in the setting of early-stage NSCLC.

The limitations of our study are its retrospective nature, the absence of molecular profiling and the relatively small cohort size, which may have limited the statistical power necessary to demonstrate significant prognostic value concerning recurrence, despite the observed statistically significance in overall survival. Additionally, we did not conduct any further immunohistochemistry analysis to identify the differential expression of PD-1 in the diverse subsets of immune cells in the tissue. We strived to account for tumor heterogeneity by having two TMA cores from each tumor (17). The staining was manually scored, which may cause a higher margin of error, and we sought to overcome this by evaluating a larger cohort. It is possible to use image analysis software, as well as additional immunohistochemical markers for T-cells to quantify the TILs for a baseline level to further stratify high- and low-density TILs. However, we argue that staining with additional single IHC markers will not enhance the evaluation of PD-1 positivity ratio in TILs in single PD-1 stained slides and a double staining protocol may interfere with the performance of the PD-1 antibody. Hence, the identification of TILs was based on morphology. Our goal was to recreate real life conditions in a routine diagnostic laboratory setting.

We intend to further explore the dynamics between PD-1, PD-L1, TILs, and other variables involved in the anti-tumoral immune response, recognizing the potential for PD-1 to be included in routine IHC panels for NSCLC diagnostics as a prognostic and possibly a co-predictive marker for ICI treatment.

Our future directions will focus on investigating the dynamic between PD-1 and PD-L1 by using proximity ligation assays (PLA). Additionally, we aim to broaden the scope of our research by identifying key T-cell subsets and other components of the immune microenvironment, as well as by molecularly characterizing markers implicated in the PD-1/PD-L1 pathway.

## Conclusion

High PD-1 expression in TILs of ≥10% in early-stage NSCLC is associated with worse prognosis of 10-year OS. These observations suggest that increased PD-1 expression in TILs may correlate with tumor progression and could prove as a potential prognostic biomarker in early-stage NSCLC.

## Data Availability

The raw data supporting the conclusions of this article will be made available by the authors, without undue reservation.

## References

[1] Cancer Today (2024). Available online at: https://gco.iarc.who.int/today/ (March 15, 2024).

[2] Mamdani H Matosevic S Khalid AB Durm G Jalal SI. Immunotherapy in lung cancer: current landscape and future directions. Front Immunol. (2022) 13:823618. doi: 10.3389/fimmu.2022.823618 35222404 PMC8864096

[3] Boussiotis VA. Molecular and biochemical aspects of the PD-1 checkpoint pathway. N Engl J Med. (2016) 375:1767–78. doi: 10.1056/NEJMra1514296 PMC5575761 27806234

[4] Francisco LM Sage PT Sharpe AH. The PD-1 pathway in tolerance and autoimmunity. Immunol Rev. (2010) 236:219–42. doi: 10.1111/j.1600-065X.2010.00923.x PMC2919275 20636820

[5] Hanahan D Weinberg RA. Hallmarks of cancer: the next generation. Cell. (2011) 144:646–74. doi: 10.1016/j.cell.2011.02.013 21376230

[6] Chen DS Mellman I. Oncology meets immunology: the cancer-immunity cycle. Immunity. (2013) 39:1–10. doi: 10.1016/j.immuni.2013.07.012 23890059

[7] Quail DF Joyce JA. Microenvironmental regulation of tumor progression and metastasis. Nat Med. (2013) 19:1423–37. doi: 10.1038/nm.3394 PMC3954707 24202395

[8] Borghaei H Paz-Ares L Horn L Spigel DR Steins M Ready NE. Nivolumab versus docetaxel in advanced nonsquamous non-small-cell lung cancer. N Engl J Med. (2015) 373:1627–39. doi: 10.1056/NEJMoa1507643 PMC5705936 26412456

[9] Brahmer J Reckamp KL Baas P Crinò L Eberhardt WEE Poddubskaya E. Nivolumab versus docetaxel in advanced squamous-cell non-small-cell lung cancer. N Engl J Med. (2015) 373:123–35. doi: 10.1056/NEJMoa1504627 PMC4681400 26028407

[10] Reck M Rodríguez-Abreu D Robinson AG Hui R Csőszi T Fülöp A. Pembrolizumab versus chemotherapy for PD-L1-positive non-small-cell lung cancer. N Engl J Med. (2016) 375:1823–33. doi: 10.1056/NEJMoa1606774 27718847

[11] Garon EB Rizvi NA Hui R Leighl N Balmanoukian AS Eder JP. Pembrolizumab for the treatment of non-small-cell lung cancer. N Engl J Med. (2015) 372:2018–28. doi: 10.1056/NEJMoa1501824 25891174

[12] Rittmeyer A Barlesi F Waterkamp D Park K Ciardiello F von Pawel J. Atezolizumab versus docetaxel in patients with previously treated non-small-cell lung cancer (OAK): a phase 3, open-label, multicentre randomised controlled trial. Lancet. (2017) 389:255–65. doi: 10.1016/S0140-6736(16)32517-X PMC6886121 27979383

[13] Schmidt LH Kümmel A Görlich D Mohr M Bröckling S Mikesch JH. PD-1 and PD-L1 expression in NSCLC indicate a favorable prognosis in defined subgroups. PloS One. (2015) 10:e0136023. doi: 10.1371/journal.pone.0136023 26313362 PMC4552388

[14] Montero MA Aricak O Kis L Yoshikawa A De Petris L Grundberg O. Clinicopathological significance of the expression of PD-L1 in non-small cell lung cancer. Ann Diagn Pathol. (2021) 51:151701. doi: 10.1016/j.anndiagpath.2021.151701 33485052

[15] Amin MB Greene FL Edge SB Compton CC Gershenwald JE Brookland RK. The Eighth Edition AJCC Cancer Staging Manual: Continuing to build a bridge from a population-based to a more “personalized” approach to cancer staging. CA Cancer J Clin. (2017) 67:93–9. doi: 10.3322/caac.21388 28094848

[16] Travis WD Brambilla E Nicholson AG Yatabe Y Austin JHM Beasley MB. The 2015 world health organization classification of lung tumors: impact of genetic, clinical and radiologic advances since the 2004 classification. J Thorac Oncol. (2015) 10:1243–60. doi: 10.1097/JTO.0000000000000630 26291008

[17] Naito T Udagawa H Sato J Horinouchi H Murakami S Goto Y. A minimum of 100 tumor cells in a single biopsy sample is required to assess programmed cell death ligand 1 expression in predicting patient response to nivolumab treatment in nonsquamous non-small cell lung carcinoma. J Thorac Oncol. (2019) 14:1818–27. doi: 10.1016/j.jtho.2019.06.019 31260834

[18] Ishida Y Agata Y Shibahara K Honjo T. Induced expression of PD-1, a novel member of the immunoglobulin gene superfamily, upon programmed cell death. EMBO J. (1992) 11:3887–95. doi: 10.1002/j.1460-2075.1992.tb05481.x PMC556898 1396582

[19] Yoshida T Jiang F Honjo T Okazaki T. PD-1 deficiency reveals various tissue-specific autoimmunity by H-2b and dose-dependent requirement of H-2g7 for diabetes in NOD mice. Proc Natl Acad Sci U S A. (2008) 105:3533–8. doi: 10.1073/pnas.0710951105 PMC2265199 18299579

[20] Okazaki T Honjo T. PD-1 and PD-1 ligands: from discovery to clinical application. Int Immunol. (2007) 19:813–24. doi: 10.1093/intimm/dxm057 17606980

[21] Juneja VR McGuire KA Manguso RT LaFleur MW Collins N Haining WN. PD-L1 on tumor cells is sufficient for immune evasion in immunogenic tumors and inhibits CD8 T cell cytotoxicity. J Exp Med. (2017) 214:895–904. doi: 10.1084/jem.20160801 28302645 PMC5379970

[22] Schalper KA Brown J Carvajal-Hausdorf D McLaughlin J Velcheti V Syrigos KN. Objective measurement and clinical significance of TILs in non–small cell lung cancer. JNCI: J Natl Cancer Institute. (2015) 107:dju435. doi: 10.1093/jnci/dju435 PMC4565530 25650315

[23] Banchereau R Chitre AS Scherl A Wu TD Patil NS de AP. Intratumoral CD103+ CD8+ T cells predict response to PD-L1 blockade. J Immunother Cancer. (2021) 9:e002231. doi: 10.1136/jitc-2020-002231 33827905 PMC8032254

[24] Edlund K Madjar K Mattsson JSM Djureinovic D Lindskog C Brunnström H. Prognostic impact of tumor cell programmed death ligand 1 expression and immune cell infiltration in NSCLC. J Thorac Oncol. (2019) 14:628–40. doi: 10.1016/j.jtho.2018.12.022 30639618

[25] Schulze AB Evers G Görlich D Mohr M Marra A Hillejan L. Tumor infiltrating T cells influence prognosis in stage I-III non-small cell lung cancer. J Thorac Dis. (2020) 12:1824–42. doi: 10.21037/jtd-19-3414a PMC7330340 32642087

[26] Donnem T Hald SM Paulsen EE Richardsen E Al-Saad S Kilvaer TK. Stromal CD8+ T-cell density—A promising supplement to TNM staging in non-small cell lung cancer. Clin Cancer Res. (2015) 21:2635–43. doi: 10.1158/1078-0432.CCR-14-1905 25680376

[27] Al-Shibli K Al-Saad S Andersen S Donnem T Bremnes RM Busund LT. The prognostic value of intraepithelial and stromal CD3-, CD117- and CD138-positive cells in non-small cell lung carcinoma. APMIS. (2010) 118:371–82. doi: 10.1111/j.1600-0463.2010.02609.x 20477813

[28] Muenst S Hoeller S Dirnhofer S Tzankov A. Increased programmed death-1+ tumor-infiltrating lymphocytes in classical Hodgkin lymphoma substantiate reduced overall survival. Hum Pathol. (2009) 40:1715–22. doi: 10.1016/j.humpath.2009.03.025 19695683

[29] Carreras J Lopez-Guillermo A Roncador G Villamor N Colomo L Martinez A. High numbers of tumor-infiltrating programmed cell death 1-positive regulatory lymphocytes are associated with improved overall survival in follicular lymphoma. J Clin Oncol. (2009) 27:1470–6. doi: 10.1200/JCO.2008.18.0513 19224853

[30] Pollari M Brück O Pellinen T Vähämurto P Karjalainen-Lindsberg ML Mannisto S. PD-L1+ tumor-associated macrophages and PD-1+ tumor-infiltrating lymphocytes predict survival in primary testicular lymphoma. Haematologica. (2018) 103:1908–14. doi: 10.3324/haematol.2018.197194 PMC6278972 30026337

[31] Owen D Chu B Lehman AM Annamalai L Yearley JH Shilo K. Expression patterns, prognostic value, and intratumoral heterogeneity of PD-L1 and PD-1 in thymoma and thymic carcinoma. J Thorac Oncol. (2018) 13:1204–12. doi: 10.1016/j.jtho.2018.04.013 PMC7193686 29702286

[32] Paulsen EE Kilvaer TK Khanehkenari MR Al-Saad S Hald SM Andersen S. Assessing PDL-1 and PD-1 in non-small cell lung cancer: A novel immunoscore approach. Clin Lung Cancer. (2017) 18:220–233.e8. doi: 10.1016/j.cllc.2016.09.009 27816392

[33] Koh J Kim S Woo YD Song SG Yim J Han B. TCF1+PD-1+ tumour-infiltrating lymphocytes predict a favorable response and prolonged survival after immune checkpoint inhibitor therapy for non-small-cell lung cancer. Eur J Cancer. (2022) 174:10–20. doi: 10.1016/j.ejca.2022.07.004 35970031

[34] Lafuente-Sanchis A Zúñiga Á Estors M Martínez-Hernández NJ Cremades A Cuenca M. Association of PD-1, PD-L1, and CTLA-4 gene expression and clinicopathologic characteristics in patients with non-small-cell lung cancer. Clin Lung Cancer. (2017) 18:e109–16. doi: 10.1016/j.cllc.2016.09.010 27816393

[35] Deng L Gyorffy B Na F Chen B Lan J Xue J. Association of PDCD1 and CTLA-4 gene expression with clinicopathological factors and survival in non-small-cell lung cancer: results from a large and pooled microarray database. J Thorac Oncol. (2015) 10:1020–6. doi: 10.1097/JTO.0000000000000550 26134222

[36] He Y Yu H Rozeboom L Rivard CJ Ellison K Dziadziuszko R. LAG-3 protein expression in non-small cell lung cancer and its relationship with PD-1/PD-L1 and tumor-infiltrating lymphocytes. J Thorac Oncol. (2017) 12:814–23. doi: 10.1016/j.jtho.2017.01.019 28132868

[37] O’Brien M Paz-Ares L Marreaud S Dafni U Oselin K Havel L. Pembrolizumab versus placebo as adjuvant therapy for completely resected stage IB-IIIA non-small-cell lung cancer (PEARLS/KEYNOTE-091): an interim analysis of a randomised, triple-blind, phase 3 trial. Lancet Oncol. (2022) 23:1274–86. doi: 10.1016/S1470-2045(22)00518-6 36108662

[38] Felip E Altorki N Zhou C Vallières E Martínez-Martí A Rittmeyer A. Overall survival with adjuvant atezolizumab after chemotherapy in resected stage II-IIIA non-small-cell lung cancer (IMpower010): a randomised, multicentre, open-label, phase III trial. Ann Oncol. (2023) 34:907–19. doi: 10.1016/j.annonc.2023.07.001 37467930

[39] Forde PM Spicer J Lu S Provencio M Mitsudomi T Awad MM. Neoadjuvant nivolumab plus chemotherapy in resectable lung cancer. N Engl J Med. (2022) 386:1973–85. doi: 10.1056/NEJMoa2202170 PMC9844511 35403841

[40] Sánchez-Magraner L Gumuzio J Miles J Quimi N Martínez Del Prado P Abad-Villar MT. Functional engagement of the PD-1/PD-L1 complex but not PD-L1 expression is highly predictive of patient response to immunotherapy in non-small-cell lung cwancer. J Clin Oncol. (2023) 41:2561–70. doi: 10.1200/JCO.22.01748 PMC10414696 36821809

