# Supplementary material for: PD-1 expression in tumor infiltrating lymphocytes as a prognostic marker in early-stage non-small cell lung cancer
Source: Front Oncol. 2024 Sep 26;14:1414900. doi: 10.3389/fonc.2024.1414900 (PMC11464330; doi:10.3389/fonc.2024.1414900)
Supplement: Supplementary file 1 [file DataSheet1.docx]

Supplementary Material

# Supplementary Tables

| **Site of relapse^*1^** | **N = 474** | **Percentage** |
| --- | --- | --- |
| No relapse | 300 | 63% |
| Lung | 47 | 9.9% |
| CNS | 28 | 5.9% |
| Bones | 19 | 4% |
| LNs | 11 | 2.3% |
| Liver | 8 | 1.7% |
| Other*^2^ | 16 | 3.4% |
| Lung and liver | 1 | 0.2% |
| Lung and bones | 3 | 0.6% |
| Lung and LNs | 18 | 3.8% |
| Lung and CNS | 4 | 0.8% |
| Lung and other | 9 | 1.9% |
| CNS and bones | 1 | 0.2% |
| CNS and LNs | 1 | 0.2% |
| CNS and other | 1 | 0.2% |
| Bones and LNs | 1 | 0.2% |
| Bones and liver | 2 | 0.4% |
| Bones and other | 2 | 0.4% |
| Liver and LNs | 1 | 0.2% |
| LNs and other | 1 | 0.2% |

**Table S1:** Site of first relapse after surgery.

Abbreviations: CNS = Central Nervous System, LNs = Intrathoracic lymph nodes. *1 : Site of first relapse *2: Other include adrenal, peritoneal or metastases in other organ systems

# Supplementary Figures

**Supplemental Figure S1. Flowchart for patient exclusion.**

*Patients with unsatisfactory stained TMA cores had a median age of 66 years, comprising 3 males and 10 females. The cohort included 8 patients with stage I cancer and 5 with stage II cancer. Histologically, there were 6 cases of squamous cell carcinoma, 6 of adenocarcinoma, and 1 of large cell carcinoma. Smoking history revealed 6 current smokers, 2 former smokers, 2 never smokers, and 3 with missing data. The median overall survival for this group was 54 months.


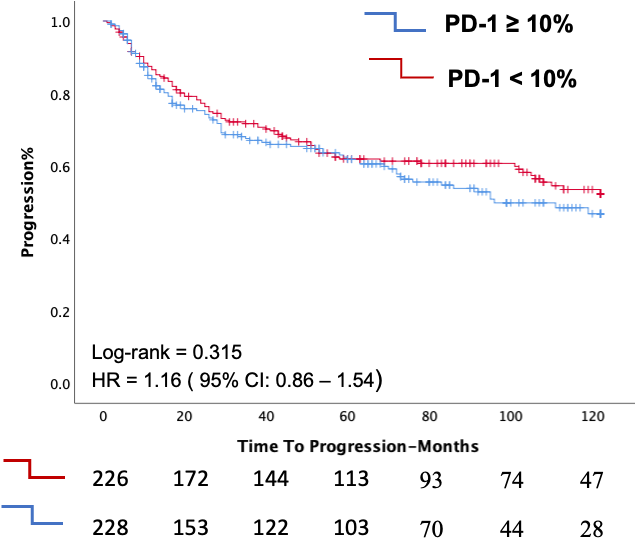


**Supplemental Figure S2:** Kaplan Meier curve demonstrating the difference on Time to Progression according to PD-1 expression in T cells by using a 10% expression cut-off.


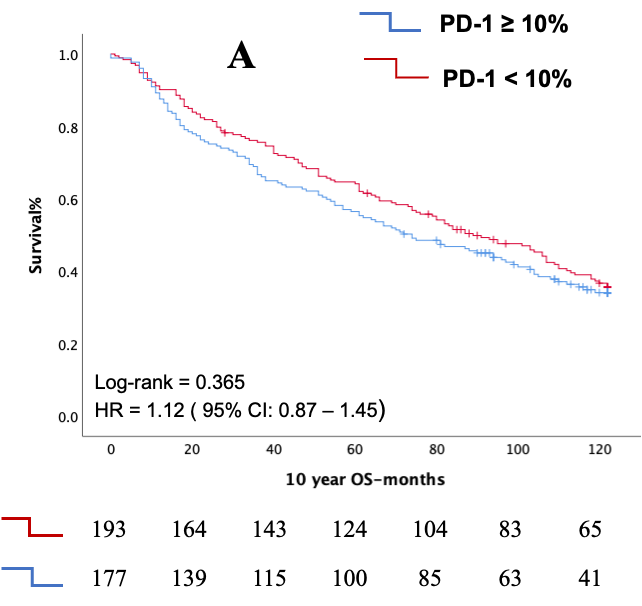

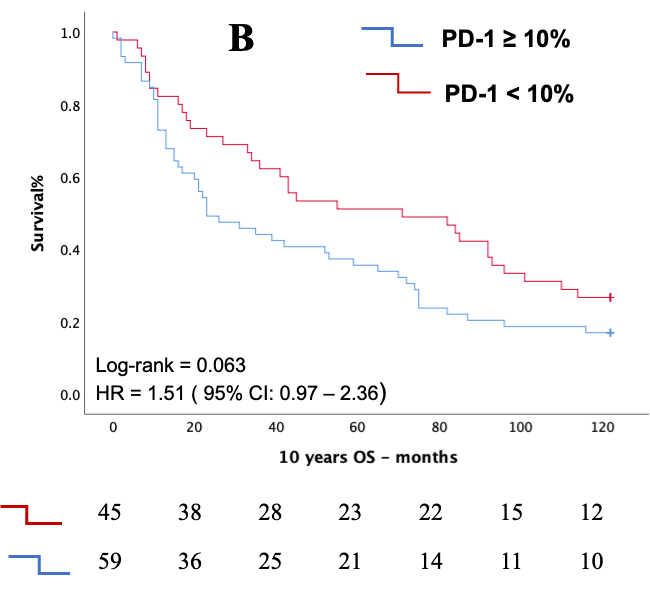


**Supplemental Figure S3:** Kaplan Meier curves depicting the effect of PD-1 expression on T cells by using a 10% cut-off for 10-year overall survival **A**. Stage I NSCLC patients in the analyzed cohort and **B**. Stage II NSCLC patients in the analyzed cohort.
